# Contribution of Pollinator-Mediated Crops to Nutrients in the Human Food Supply

**DOI:** 10.1371/journal.pone.0021363

**Published:** 2011-06-22

**Authors:** Elisabeth J. Eilers, Claire Kremen, Sarah Smith Greenleaf, Andrea K. Garber, Alexandra-Maria Klein

**Affiliations:** 1 Department of Applied Zoology/Animal Ecology, Freie Universität Berlin, Berlin, Germany; 2 Department of Evolutionary Neuroethology, Max-Planck-Institute for Chemical Ecology, Jena, Germany; 3 Environmental Sciences Policy and Management, University of California, Berkeley, California, United States of America; 4 Department of Plant Pathology, University of California Davis, Davis, California, United States of America; 5 Division of Adolescent Medicine, University of California San Francisco, San Francisco, California, United States of America; 6 Institute of Ecology, Section Ecosystem Functions, Leuphana University of Lüneburg, Lüneburg, Germany; Ghent University, Belgium

## Abstract

The contribution of nutrients from animal pollinated world crops has not previously been evaluated as a biophysical measure for the value of pollination services. This study evaluates the nutritional composition of animal-pollinated world crops. We calculated pollinator dependent and independent proportions of different nutrients of world crops, employing FAO data for crop production, USDA data for nutritional composition, and pollinator dependency data according to Klein et al. (2007). Crop plants that depend fully or partially on animal pollinators contain more than 90% of vitamin C, the whole quantity of Lycopene and almost the full quantity of the antioxidants β-cryptoxanthin and β-tocopherol, the majority of the lipid, vitamin A and related carotenoids, calcium and fluoride, and a large portion of folic acid. Ongoing pollinator decline may thus exacerbate current difficulties of providing a nutritionally adequate diet for the global human population.

## Introduction

Crop pollination mediated by wild and domesticated animals is a crucial and endangered ecosystem service [1], [2]. Recently, the global economic value of pollination from domesticated and wild animals has been estimated at €153 billion, while the consumer surplus loss associated with a total loss of animal pollination service was estimated between €190 and €310 billion [3].

The decline of pollinators has become a major problem at a time when the global demand for crop pollinators is increasing [4]. Several studies have been conducted to evaluate monetary values of pollination services on crop pollination [3] [5] [6]. However, it is difficult to assign monetary values to ecosystem services because they are frequently not traded on the marketplace [7] and values differ widely depending on methods, value systems, and scales of analysis [8] [9] [10]. Furthermore, the value of money changes constantly with shifting markets, particularly in the face of the current global financial crisis. In contrast, biophysical measures such as the nutritional composition of animal-pollinated plants and nutrient requirements to prevent deficiency in humans are relatively stable and may be measured objectively. Here we take this biophysical approach to evaluate the global nutritional value of pollinator-dependent crops.

Staple crop production (e.g. cassava, corn, potato, rice, wheat, yam) has doubled in the past 50 years due to new crop strains, increased use of agrochemicals, irrigation and new agricultural techniques [11]. These grains and starchy vegetables are mostly wind-pollinated, self-pollinated, or vegetatively propagated crops [11]. While they provide the majority of calories in the human diet [12] [13] [14], they are poor sources of most micronutrients [15]. What little micronutrients are present in these sources are mostly lost in processing or preservation [16]. Dependence on these staple crops due to food system failures and declines in diet diversity are responsible for micronutrient deficiency (‘Hidden Hunger’) in over two billion people worldwide, especially in underprivileged areas [17]. This underscores the importance of diet diversity and the need for animal-pollinated plants to prevent micronutrient deficiency. However the contribution of these plants to worldwide micronutrient availability has not been quantified. Therefore, the potential impact of pollinator decline on global health and nutrition is difficult to estimate. Any impact is likely to be more dramatic in developing countries, which are already vulnerable to food and nutrient shortages related to demographic and climate change [18].

## Materials and Methods

We selected FAO data on the worldwide production of more than 150 leading global crops from 1997 to 2007 [19] to calculate the respective annual mean production for each crop. Nutrient content data on these raw crops and percentage of refuse were obtained from the USDA database [20]. Total energy (in joules), macronutrients (carbohydrate, protein and fat) and micronutrients were analyzed. Micronutrient analysis included minerals (calcium, iron, magnesium, phosphorus, potassium, sodium, zinc, copper, manganese, selenium, and fluoride), water-soluble vitamins (vitamin C, thiamine, riboflavin, niacin, vitamin B5, vitamin B6, folic acid), and fat-soluble vitamins [E (including β-, δ-, and γ- tocopherol), K, A and related carotenoids (including α- and β- carotene, β- cryptoxanthin, lycopene, lutein, and zeaxanthin)]. Spices, condiments, stimulant crops, sugars (from cane and beet), and processed and animal-derived products were excluded from our analysis. Food crop production was considered pollinator-independent for our calculations for crops in which animal pollination increased seed production (e.g. in onion and asparagus), but did not directly result in an increase of edible plant material.

Proportion of crop production depending on animal pollination was derived according to estimates by Klein et al. (2007). Based on the obtained data, the proportion of pollinator-independent nutritional values was calculated, applying the following equation, summed over each crop type:
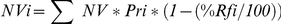
The proportion of nutritional values derived from pollinator-dependent crops was calculated, applying the following equation:
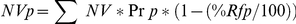
Since there is a spectrum of pollinator-dependence categories among pollinator-dependent crops, ranging from no = 0%, little = 5%, modest = 25%, great = 65%, to an essential >65% impact of pollinators to production [2], this proportion was further divided into the actual increase due to animal pollinators (pollinator-dependent component) versus the remaining pollinator-independent component, subject to the plant's degree of animal pollination dependency:
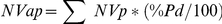


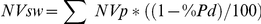



NVi = Nutrients derived from completely pollinator-independent crop production (all autonomous self or wind pollination)

NVp = Nutrients derived from pollinator-dependent crop production (including proportions attributed to animal pollination and to autonomous self and wind pollination)

NVap = Nutrients derived from crop production attributed to animal pollination alone

NVsw = Nutrients derived from crop production for partially animal-dependent crops attributed to autonomous self- or wind pollination

NV = Amount of nutritional component in crop production (in metric tons)

Pr = Average production 1997–2007 of crop production (in metric tons)

Rf = Percent refuse, e.g. pits, stems, or shells.

Pd = Proportion of crop production in pollinator dependence categories according to Klein et al. (2007), no impact = 0%, little = 5%, modest = 25%, great = 65%, essential = 95%.

## Results and Discussion

We examined the nutrient availability in more than 150 of the world's leading crops and found that the majority of the lipid and several micronutrients required for human health are present in plants that are animal pollinated.

**Figure 1:**
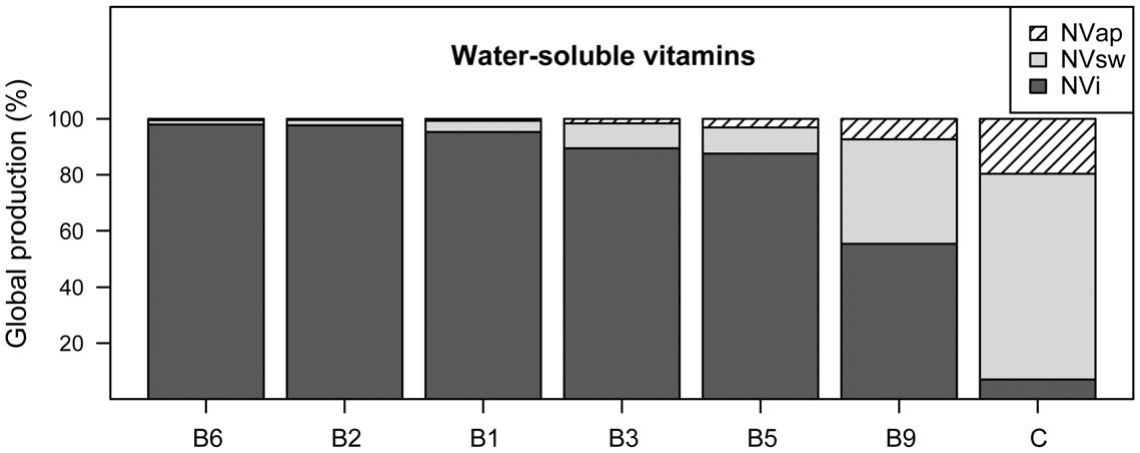
Proportion of water-soluble vitamins (B6 = vitamin B6, B2 = riboflavin, B1 = thiamine, B3 = niacin, B5 = pantothenic acid, B9 = folate/folic acid, C = vitamin C) in global crop production (%) produced without pollinators (NVi, dark-grey), produced with pollinators but attributed to autonomous self- or wind pollination (NVsw, light-grey), produced with pollinators and directly attributed to animal pollination (NVap, hatched).

**Figure 2:**
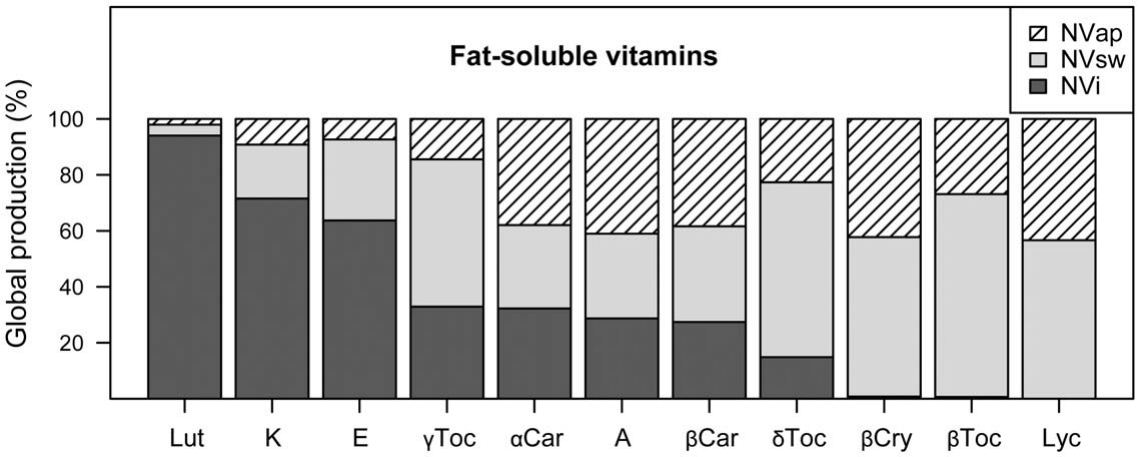
Proportion of fat-soluble vitamins (lut = lutein, K = vitamin K, E = vitamin E, γToc = γ – tocopherol, αCar = α-carotene, A = vitamin A, βCar = β-carotene, δToc = δ – tocopherol, βCry = β - cryptoxanthin, βToc = β – tocopherol, Lyc = lycopene) in global crop production (%) produced without pollinators (NVi, dark-grey), produced with pollinators but attributed to autonomous self- or wind pollination (NVsw, light-grey), produced with pollinators and directly attributed to animal pollination (NVap, hatched).

According to our estimates, around 74% of all globally produced lipids are present in oils from plants that are promoted by animal pollination (Table 1); these plants also serve as primary sources of the fat-soluble vitamins. Of the water-soluble vitamins, 98% of the available vitamin C comes from animal-pollinated plants, primary citrus and other fruits and vegetables (Fig. 1, Table S1). While scurvy due to vitamin C deficiency is now uncommon, the antioxidant role of vitamin C, along with vitamin E and β-carotene, is well recognized [21]. The water-soluble B vitamins are abundant in starchy staple crops that propagate independently of pollinator deficiency (Table S1). However, the majority of these nutrients are lost when whole grains are processed into the refined starches that are preferred globally (e.g. white rice and white flour). While the U.S. has rectified this loss by heavily fortifying refined wheat flours, 2/3 of the population globally does not have access to fortified grains [22]. This underscores the importance of plant sources of the B vitamins, particularly folic acid. The requirement for folic acid is increased during pregnancy to prevent neural tube defects in the infant [16]. We found that globally 55% of available folate is present in crop plants that are animal pollinated, including beans and dark green leafy vegetables (Table S1), with a 7.3% direct yield increase due to animal pollination. More than 70% of vitamin A and 98% of each of the carotenoids cryptoxanthin (provitamin A) and lycopene are found in crops that are animal-pollinated (Table 1, Fig. 2). It is not known to what extent these plants, including red, orange and yellow vegetables and fruits may propagate without animal pollination, but the direct yield increase due to pollination has been estimated at 43%. Vitamin A is one of the most prevalent deficiencies worldwide and is responsible for up to 500,000 annual cases of irreversible blindness in children worldwide [15]. Diets high in carotenoids are protective against cancer; lycopene has shown a suppression of tumour incidence in *in vivo* studies [23]. A large portion of the dietary vitamin E is present in plants that require pollination. The primary role of vitamin E is as an antioxidant; higher intakes are associated with lower cardiovascular disease (CVD) risk. Current human nutrient requirements are given in α-tocopherol units because it is the most absorbable form, but γ-tocopherol is the most common dietary form (mostly in the form of plant oils). We showed that more than 35% of the α- and 66% of the γ-tocopherol are present in pollinator-dependent plants, with an estimated yield increase of 14–27% (Fig. 2).

**Table 1:** Amount [in metric tons (mt) and Gigajoules (GJ)] and proportion (%) of nutrients derived from pollinator-independent crop production (NVi) and from pollinator-dependent crop production, divided into values attributed to wind- or autonomous self-pollination (NVsw) and values attributed to animal pollination (NVap).

Nutritional component		Pollinator independent		Pollinator dependent			
		NViGJ, mt	NVi%	NVswGJ, mt	NVsw%	NVapGJ, mt	NVap%
ENERGY		3.06E+10	78.83	7.217E+9	18.59	1.001E+9	2.58
MACRO-NUTRIENTS							
	Protein	2.238E+8	83.43	3.641E+7	13.57	8.060E+6	3.00
	Fat	7.363E+7	26.02	1.896E+8	66.98	1.982E+7	7.00
VITAMINS							
Vitamin A		8.549E+5	28.71	9.009E+5	30.26	1.222E+6	41.03
Carotenoids	β - carotene	1.476E+6	27.44	1.839E+6	34.19	2.064E+6	38.37
	α - carotene	4.960E+5	32.25	4.587E+5	29.83	5.832E+5	37.92
	β - cryptoxanthin	3.579E+3	0.77	2.637E+5	56.99	1.954E+5	42.24
	Lycopene	0	0	2.941E+6	56.67	2.248E+6	43.33
	Lutein, zeaxanthin	1.367E+7	94.05	5.696E+5	3.92	2.96 E+5	2.03
Vitamin E	α-tocopherol	1.72 E+7	63.73	7.811E+6	28.94	1.978E+6	7.33
	β-tocopherol	1.229E+3	0.63	1.415E+5	72.50	5.245E+4	26.87
	γ-tocopherol	5.361E+5	32.92	8.574E+5	52.66	2.349E+5	14.42
	δ-tocopherol	1.209E+4	14.87	5.081E+4	62.50	1.84 E+4	22.63
Vitamin K		8.96 E+4	71.55	2.414E+4	19.28	1.148E+4	9.17
Vitamin C		1.211E+4	6.99	1.272E+8	73.37	3.406E+7	19.64
Vitamin B	Thiamin (B1)	4.450E+7	95.29	1.866E+6	4.00	3.327E+5	0.71
	Riboflavin (B2)	3.942E+7	97.66	7.754E+5	1.92	1.702E+5	0.42
	Niacin (B3)	1.240E+8	89.46	1.238E+7	8.93	2.231E+6	1.61
	Pantothenic acid (B5)	1.723E+7	87.57	1.839E+6	9.34	6.077E+5	3.09
	Vitamin B6	4.784E+7	97.93	7.701E+5	1.58	2.383E+5	0.49
	Folate, total (B9)	7.114E+5	55.49	4.767E+5	37.19	9.389E+4	7.32
MINERALS							
Calcium		5.245E+5	42.40	5.998E+5	48.49	1.127E+5	9.11
Iron		6.537E+4	70.66	2.141E+4	23.14	5.741E+3	6.20
Magnesium		2.7 E+6	88.50	2.765E+5	9.06	7.433E+4	2.44
Phosphorus		6.589E+6	89.06	6.448E+5	8.72	1.644E+5	2.22
Potassium		7.327E+6	72.74	2.109E+6	20.93	6.372E+5	6.33
Sodium		4.703E+5	87.18	4.654E+4	8.63	2.259E+4	4.19
Zinc		6.658E+4	91.80	4.745E+3	6.54	1.201E+3	1.66
Copper		8.048E+3	80.92	1.512E+3	15.21	3.851E+2	3.87
Mangan		5.048E+4	93.87	2.655E+3	4.94	6.387E+2	1.19
Selenium		7.86 E+5	97.46	1.590E+4	1.97	4.548E+3	0.57
Fluoride		4.962E+3	45.57	3.768E+3	34.60	2.16 E+3	19.83

**Figure 3:**
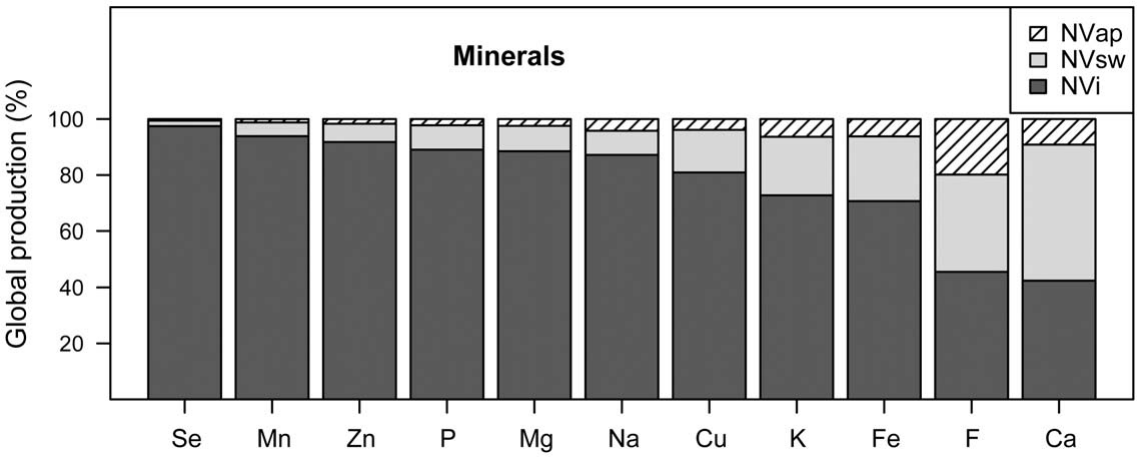
Proportion of minerals (Se = selenium, Mn = manganese, Zn = zinc, P = phosphorus, Mg = magnesium, Na = sodium, Cu = copper, K = potassium, Fe = iron, F = fluoride and Ca = calcium) in global crop production (%) produced without pollinators (NVi, dark-grey), produced with pollinators but attributed to autonomous self- or wind pollination (NVsw, light-grey), produced with pollinators and directly attributed to animal pollination (NVap, hatched).

Animal pollinated plants are also important contributors of minerals available in the human diet. Fifty-eight percent of calcium and 62% of fluoride are derived from plants with marginally yield increase due to animal pollination, such as beans, but also strongly pollinator dependent plants such as fruits and nuts (including almond)(Table 1, Fig. 3). The respective proportion directly derived due to yield increase after pollination is 9% for calcium and 20% for fluoride. These minerals are crucial for development of teeth and bones and prevention of age-related bone loss that contributes to osteoporotic fracture risk [24] [25]. Animal sources of calcium (such as dairy) are more bioavailable than plant sources. However, dairy production is costly and environmentally inefficient and consumption is not culturally feasible on a global scale [26]. Similarly, iron from animal sources (meat) is more bioavailable than plant sources but costly and inefficient [27]. Iron deficiency is thought to be the most common micronutrient deficiency, contributing heavily to preventable cognitive impairment and infection worldwide [17], [28], [29]. Thus, plant sources of iron are crucial to human health and we found that 29% of iron is derived from pollinator-dependent crops, with 6% yield increase due to animal pollination (Table 1, Fig. 3).

We recognize that our findings are limited by the use of nutrient analysis data generated in the U.S. The USDA Nutrient Data Laboratory is recognized as an authoritative source of nutrient analysis data that is used extensively in dietary and health policy and research in the U.S [20]. However, the foods analyzed are likely (but not exclusively) grown in U.S. and may not represent the nutritional content of the same food grown elsewhere. The FAO has recognized this limitation and has developed a nutrient database directory that offers preliminary searchable and comprehensive data since December 2010 [30]. Fortunately, regional variation in nutrient content is thought to be more strongly related to processing than to growing conditions, and we did not include processed foods in this analysis. Moreover, our study did not consider spatial heterogeneity of pollinator declines and variation in production and consumption of pollinator dependent crops among regions. Several crops are consumed in affluent countries with known pollinator declines for both managed and unmanaged pollinators (e.g. the U.S. and many European countries) [19], but are largely produced in other countries, for example watermelon (75% of global production in China), apples (50% of global production in China) and mangoes (42% of global production in India). Conversely, 75% of the global production of almonds is in one region of the United States (California) m of which 80% is exported [31]. Unfortunately, to date only a few regional studies have been conducted on pollinator declines [32], [33] and thus it is not currently possible to determine how variation in pollinator declines among regions of the world may affect the production of specific commodities (particularly those largely produced within a particular region), as well as their consumption in other parts of the world.

We conclude based on this analysis that animal-pollinated crops contain the majority of the available dietary lipid, vitamin A, C and E, and a large portion of the minerals calcium, fluoride, and iron worldwide. The yield increase attributable to animal-dependent pollination of these crops is significant and could have a potentially drastic effect on human nutrition if jeopardized.

Micronutrient deficiencies resulting from potential declines in animal-pollinated crops are likely to be worse in developing nations. Supplementation and fortification are not adequate substitutes for the loss or reduction of these nutrients from food sources. Mandatory fortification has been successful in some countries, such as the U.S. and China, but it depends on an organized and regulated food industry. Supplementation is not globally feasible for several reasons. First, it depends on the affluence and educational level of the consumer. Whereas the majority of adult US citizens are currently consuming vitamins, herbal drugs, and other dietary supplements [34], people in less developed countries have limited access to such components. Synthetically fabricated healthcare products are only available to 25% of the world population, while the other 75% relies on ethnobotanical remedies [35]. Second, it is likely that the plants used to develop supplements and remedies require pollination services themselves. Ninety percent of flowering tropical plant species are animal-pollinator dependent [36] and many supplements are derived from the flowering, pollinator-dependent plants. Thus, the costs of fortification and supplementation would be further increased by shrinking pollination services. Third, adequate nutrient substitutes may not exist. Much remains to be learned regarding the role of other constituent plant components in human nutrition. As yet unknown beneficial components within fruits and vegetables may explain why diets high in fruits and vegetables are associated with lower risk of cardiovascular disease and certain types of cancer, whereas no beneficial effect of supplements has been shown [37]. For example, a much anticipated large-scale trial of folate supplementation and CVD including data from nearly 17̇000 participants just found no difference between treatment and control group [38].

Finally, biofortification, the genetic engineering of crops to include vitamins [15], [16], has been plagued by difficulties, particularly for iron and other rapidly oxidizing micronutrients [16]. Collectively, these barriers suggest that supplementation and fortification are unlikely to compensate for a potential decrease in supply of essential nutrients due to pollinator decline. In conclusion, the results of this study strengthen earlier reports on the economic value of pollination services by quantifying the value to nutrition and highlighting the importance of pollinators to global health.

## Supporting Information

Table S1 **Crops containing highest proportion of nutritional components and crops with highest global production of nutritional components. Aggregated commodities:** Beans, dry: Black bean, Kidney bean, Navy bean, Northern bean. Beans, green: Black bean, Kidney bean, Navy bean, Northern bean. Fruit Fresh nes: Azarole (Azzeruolo), Babaco, Elderberry, Jujube, Litchi, Loquat (Japanese plum), Medlar, Pawpaw, Pomegranate, Prickly pear, Rose hips, Dogroses, Rowanberry, Service-apple, Tamarind. Fruit Tropical Fresh nes: Breadfruit, Carambola, Cherimoya, Durian, Feijoa, Guava, Hog-plum (yellow Mombin), Jackfruit, Longan (Lungan), Mammee, Mangosteen, Naranjillo, Passionfruit, Rambutan, Sapodilla, Sapote (marmelade plum), Star apple (Cainito). Nuts nes: Acorn, Beechnut, Butternut, Ginkgo nut, Hickorynut, Macadamia, Pecan, Pilinut, Pine nut. Pulses nes: Guar bean, Goa bean, Hyacinth bean, Horse-gram, Lablab, Jack bean, Horse bean, Sword bean, Velvet bean, Winged bean, Goa bean, Yam bean. String beans: Green snap bean, Yardlong bean, Yellow bean.
(DOC)
