# Supplementary material for: Contribution of Pollinator-Mediated Crops to Nutrients in the Human Food Supply
Source: PLoS One. 2011 Jun 22;6(6):e21363. doi: 10.1371/journal.pone.0021363 (PMC3120884; doi:10.1371/journal.pone.0021363)
Supplement: Table S1 — Crops containing highest proportion of nutritional components and crops with highest global production of nutritional components. Aggregated commodities: Beans, dry: Black bean, Kidney bean, Navy bean, Northern bean. Beans, green: Black bean, Kidney bean, Navy bean, Northern bean. Fruit Fresh nes: Azarole (Azzeruolo), Babaco, Elderberry, Jujube, Litchi, Loquat (Japanese plum), Medlar, Pawpaw, Pomegranate, Prickly pear, Rose hips, Dogroses, Rowanberry, Service-apple, Tamarind. Fruit Tropical Fresh nes: Breadfruit, Carambola, Cherimoya, Durian, Feijoa, Guava, Hog-plum (yellow Mombin), Jackfruit, Longan (Lungan), Mammee, Mangosteen, Naranjillo, Passionfruit, Rambutan, Sapodilla, Sapote (marmelade plum), Star apple (Cainito). Nuts nes: Acorn, Beechnut, Butternut, Ginkgo nut, Hickorynut, Macadamia, Pecan, Pilinut, Pine nut. Pulses nes: Guar bean, Goa bean, Hyacinth bean, Horse-gram, Lablab, Jack bean, Horse bean, Sword bean, Velvet bean, Winged bean, Goa bean, Yam bean. String beans: Green snap bean, Yardlong bean, Yellow bean. (DOC) [file pone.0021363.s001.doc]

| Nutritional component | Crops with highest content of component | Contained component per 100 g | Yield increase due to pollination (%) | Crops with highest global production of component | Yield increase due to pollination  (%) |
| --- | --- | --- | --- | --- | --- |
| Energy (kj) | Oil palm fruit | 3123.0 | 5 | Maize | 0 |
|  | Brazil Nuts | 2743.0 | 95 | Wheat | 0 |
|  | Walnuts | 2738.0 | 0 | Rice | 0 |
|  | Hazelnuts in shell | 2629.0 | 0 | Oil Palm Fruit | 5 |
|  | Sunflowerseed | 2445.0 | 25 | Barley | 0 |
|  | Nuts nes | 2424.1 | 18.33 | Sorghum | 0 |
|  | Almonds, with shell | 2408.0 | 65 | Groundnuts in Shell | 5 |
|  | Sesameseed | 2397.0 | 25 | Soybeans | 25 |
|  | Groundnuts in shell | 2374.0 | 5 | Millet | 0 |
|  | Pistachios | 2332.0 | 0 | Oats | 0 |
|  | Melonseed | 2330.0 | 95 | Coconuts | 25 |
|  | Cashew nuts, with shell | 2314.0 | 65 | Sunflowerseed | 25 |
|  | Safflowerseed | 2163.0 | 5 | Beans, dry | 5 |
|  | Oats | 1628.0 | 0 | Rye | 0 |
|  | Millet | 1582.0 | 0 | Grapes | 0 |
|  | Quinoa | 1539.0 | 0 | Triticale | 0 |
|  | Rice | 1531.5 | 0 | Peas, dry | 0 |
|  | Maize | 1527.0 | 0 | Chick-Peas | 0 |
|  | Chick-Peas | 1525.0 | 0 | Apples | 65 |
|  | Coconuts | 1481.0 | 25 | Oranges | 5 |
| Protein (g) | Melonseed | 28.33 | 95 | Wheat | 0 |
|  | Broad Beans, dry | 26.12 | 5 | Maize | 0 |
|  | Groundnuts in shell | 25.80 | 5 | Rice | 0 |
|  | Lentils | 25.80 | 0 | Barley | 0 |
|  | Peas, dry | 24.55 | 0 | Soybeans | 25 |
|  | Cow Peas, dry | 23.52 | 5 | Groundnuts in Shell | 5 |
|  | Beans, green | 23.34 | 5 | Sorghum | 0 |
|  | Beans, dry | 22.34 | 5 | Oats | 0 |
|  | Pulses nes | 21.82 | 5 | Beans, dry | 5 |
|  | Pigeon Peas | 21.70 | 5 | Millet | 0 |
|  | Almonds, with shell | 21.22 | 65 | Oil Palm Fruit | 5 |
|  | Sunflowerseed | 20.78 | 25 | Sunflowerseed | 25 |
|  | Pistachios | 20.61 | 0 | Rye | 0 |
|  | Chick-Peas | 19.30 | 0 | Peas, dry | 0 |
|  | Cashew nuts, with shell | 18.22 | 65 | Chick-Peas | 0 |
|  | Sesameseed | 17.73 | 25 | Triticale | 0 |
|  | Oats | 16.89 | 0 | Beans, Green | 5 |
|  | Safflowerseed | 16.18 | 5 | Broad Beans, dry | 5 |
|  | Walnuts | 15.23 | 0 | Cow Peas, dry | 5 |
|  | Hazelnuts in shell | 14.95 | 0 | Coconuts | 25 |
| Lipid (g) | Oil palm fruit | 81.90 | 5 | Oil palm fruit | 5 |
|  | Brazil Nuts | 66.43 | 95 | Maize | 0 |
|  | Walnuts | 65.21 | 0 | Coconuts | 25 |
|  | Hazelnuts in shell | 60.75 | 0 | Groundnuts in shell | 5 |
|  | Nuts nes | 54.73 | 18.33 | Rice | 0 |
|  | Sunflowerseed | 51.46 | 25 | Sunflowerseed | 25 |
|  | Sesameseed | 49.67 | 25 | Soybeans | 25 |
|  | Almonds, with shell | 49.42 | 65 | Wheat | 0 |
|  | Groundnuts in shell | 49.24 | 5 | Peaches and nectarines | 65 |
|  | Melonseed | 47.37 | 95 | Sorghum | 0 |
|  | Pistachios | 44.44 | 0 | Oats | 0 |
|  | Cashew nuts, with shell | 43.85 | 65 | Olives | 0 |
|  | Safflowerseed | 38.45 | 5 | Barley | 0 |
|  | Coconuts | 33.49 | 25 | Sesameseed | 25 |
|  | Peaches and nectarines | 28.50 | 65 | Millet | 0 |
|  | Avocados | 15.41 | 65 | Cashew nuts, with shell | 65 |
|  | Olives | 10.68 | 0 | Walnuts | 0 |
|  | Oats | 6.90 | 0 | Almonds, with shell | 65 |
|  | Soybeans | 6.80 | 25 | Chick-Peas | 0 |
|  | Pulses nes | 6.21 | 5 | Hazelnuts in shell | 0 |
| Calcium (mg) | Sesameseed | 975.00 | 25 | Soybeans | 25 |
|  | Almonds, with shell | 264.00 | 65 | Oil palm fruit | 5 |
|  | Pulses nes | 216.93 | 5 | Wheat | 0 |
|  | Soybeans | 197.00 | 25 | Rice | 0 |
|  | Brazil Nuts | 160.00 | 95 | Maize | 0 |
|  | Beans, dry | 147.00 | 5 | Barley | 0 |
|  | Beans, green | 147.00 | 5 | Groundnuts in shell | 5 |
|  | Oil palm fruit | 136.10 | 5 | Sesameseed | 25 |
|  | Pigeon Peas | 130.00 | 5 | Beans, dry | 5 |
|  | Hazelnuts in shell | 114.00 | 0 | Oranges | 5 |
|  | Cow Peas, dry | 110.00 | 5 | Sunflowerseed | 25 |
|  | Pistachios | 107.00 | 0 | Sorghum | 0 |
|  | Chick-Peas | 105.00 | 0 | Olives | 0 |
|  | Broad Beans, dry | 103.00 | 5 | Oats | 0 |
|  | Spinach | 99.00 | 0 | Tomatoes | 5 |
|  | Walnuts | 98.00 | 0 | Spinach | 0 |
|  | Groundnuts in shell | 92.00 | 5 | Beans, green | 5 |
|  | Olives | 88.00 | 0 | Chick-Peas | 0 |
|  | String beans | 84.33 | 5 | Fruit Fresh nes | 35.38 |
|  | Okra | 81.00 | 25 | Carrots & Turnips | 65 |
| Iron (mg) | Peaches and nectarines | 26.50 | 65 | Wheat | 0 |
|  | Sesameseed | 14.55 | 25 | Maize | 0 |
|  | Cow Peas, dry | 8.27 | 5 | Rice | 0 |
|  | Lentils | 7.54 | 0 | Oil palm fruit | 5 |
|  | Melonseed | 7.28 | 95 | Soybeans | 25 |
|  | Broad Beans, dry | 6.70 | 5 | Peaches and nectarines | 65 |
|  | Cashew nuts, with shell | 6.68 | 65 | Barley | 0 |
|  | Pulses nes | 6.42 | 5 | Sorghum | 0 |
|  | Chick-Peas | 6.24 | 0 | Groundnuts in shell | 5 |
|  | Beans, dry | 6.05 | 5 | Sunflowerseed | 25 |
|  | Beans, green | 6.05 | 5 | Coconuts | 25 |
|  | Chillies and peppers, dry | 6.04 | 5 | Oats | 0 |
|  | Oil palm fruit | 5.60 | 5 | Beans, dry | 5 |
|  | Sunflowerseed | 5.25 | 25 | Millet | 0 |
|  | Pigeon Peas | 5.23 | 5 | Olives | 0 |
|  | Safflowerseed | 4.90 | 5 | Chick-Peas | 0 |
|  | Oats | 4.72 | 0 | Rye | 0 |
|  | Hazelnuts in shell | 4.70 | 0 | Peas, dry | 0 |
|  | Groundnuts in shell | 4.58 | 5 | Sesameseed | 25 |
|  | Quinoa | 4.57 | 0 | Beans, green | 5 |
| Magnesium (mg) | Melonseed | 515.00 | 95 | Rice | 0 |
|  | Brazil Nuts | 376.00 | 95 | Maize | 0 |
|  | Safflowerseed | 353.00 | 5 | Wheat | 0 |
|  | Sesameseed | 351.00 | 25 | Soybeans | 25 |
|  | Sunflowerseed | 325.00 | 25 | Barley | 0 |
|  | Cashew nuts, with shell | 292.00 | 65 | Sunflowerseed | 25 |
|  | Almonds, with shell | 268.00 | 65 | Groundnuts in shell | 5 |
|  | Buckwheat | 231.00 | 65 | Oats | 0 |
|  | Quinoa | 197.00 | 0 | Millet | 0 |
|  | Broad Beans, dry | 192.00 | 5 | Beans, dry | 5 |
|  | Cow Peas, dry | 184.00 | 5 | Rye | 0 |
|  | Pigeon Peas | 183.00 | 5 | Coconuts | 25 |
|  | Oats | 177.00 | 0 | Triticale | 0 |
|  | Beans, dry | 168.75 | 5 | Tomatoes | 5 |
|  | Beans, green | 168.75 | 5 | Peas, dry | 0 |
|  | Groundnuts in shell | 168.00 | 5 | Sesameseed | 25 |
|  | Hazelnuts in shell | 163.00 | 0 | Beans, green | 5 |
|  | Walnuts | 158.00 | 0 | Chick-Peas | 0 |
|  | Nuts nes | 144.78 | 18.33 | Spinach | 0 |
|  | Rice | 143.00 | 0 | Watermelons | 95 |
| Phosphorus (mg) | Melonseed | 755.00 | 95 | Wheat | 0 |
|  | Brazil Nuts | 725.00 | 95 | Rice | 0 |
|  | Sunflowerseed | 660.00 | 25 | Maize | 0 |
|  | Safflowerseed | 644.00 | 5 | Soybeans | 25 |
|  | Sesameseed | 629.00 | 25 | Barley | 0 |
|  | Cashew nuts, with shell | 593.00 | 65 | Sunflowerseed | 25 |
|  | Oats | 523.00 | 0 | Sorghum | 0 |
|  | Pistachios | 490.00 | 0 | Oats | 0 |
|  | Almonds, with shell | 484.00 | 65 | Groundnuts in shell | 5 |
|  | Quinoa | 457.00 | 0 | Millet | 0 |
|  | Lentils | 451.00 | 0 | Beans, dry | 5 |
|  | Cow Peas, dry | 424.00 | 5 | Rye | 0 |
|  | Broad Beans, dry | 421.00 | 5 | Coconuts | 25 |
|  | Wheat | 409.20 | 0 | Triticale | 0 |
|  | Beans, dry | 403.25 | 5 | Peas, dry | 0 |
|  | Beans, green | 403.25 | 5 | Chick-Peas | 0 |
|  | Groundnuts in shell | 376.00 | 5 | Tomatoes | 5 |
|  | Rye | 374.00 | 0 | Beans, green | 5 |
|  | Pigeon Peas | 367.00 | 5 | Sesameseed | 25 |
|  | Chick-Peas | 366.00 | 0 | Broad Beans, dry | 5 |
| Potassium (mg) | Chillies and peppers, dry | 1870.00 | 5 | Wheat | 0 |
|  | Pigeon Peas | 1392.00 | 5 | Maize | 0 |
|  | Beans, dry | 1365.25 | 5 | Rice | 0 |
|  | Beans, green | 1365.25 | 5 | Soybeans | 25 |
|  | Cow Peas, dry | 1112.00 | 5 | Barley | 0 |
|  | Broad Beans, dry | 1062.00 | 5 | Tomatoes | 5 |
|  | Pistachios | 1025.00 | 0 | Beans, dry | 5 |
|  | Peas, dry | 981.00 | 0 | Groundnuts in shell | 5 |
|  | Lentils | 955.00 | 0 | Sorghum | 0 |
|  | Pulses nes | 923.11 | 5 | Coconuts | 25 |
|  | Chick-Peas | 875.00 | 0 | Sunflowerseed | 25 |
|  | Almonds, with shell | 705.00 | 65 | Grapes | 0 |
|  | Groundnuts in shell | 705.00 | 5 | Oranges | 5 |
|  | Dates | 696.00 | 0 | Oats | 0 |
|  | Safflowerseed | 687.00 | 5 | Peas, dry | 0 |
|  | Hazelnuts in shell | 680.00 | 0 | Watermelons | 95 |
|  | Cashew nuts, with shell | 660.00 | 65 | Beans, green | 5 |
|  | Brazil Nuts | 659.00 | 95 | Chillies & Peppers, green | 5 |
|  | Melonseed | 648.00 | 95 | Carrots & Turnips | 65 |
|  | Sunflowerseed | 645.00 | 25 | Chick-Peas | 0 |
| Sodium (mg) | Olives | 872.00 | 0 | Maize | 0 |
|  | Melonseed | 99.00 | 95 | Olives | 0 |
|  | Chillies and peppers, dry | 91.00 | 5 | Soybeans | 25 |
|  | Spinach | 79.00 | 0 | Carrots & Turnips | 65 |
|  | Carrots & Turnips | 69.00 | 65 | Rice | 0 |
|  | Maize | 35.00 | 0 | Barley | 0 |
|  | Chick-Peas | 24.00 | 0 | Wheat | 0 |
|  | Pulses nes | 20.11 | 5 | Coconuts | 25 |
|  | Coconuts | 20.00 | 25 | Spinach | 0 |
|  | Groundnuts in shell | 18.00 | 5 | Groundnuts in shell | 5 |
|  | Pigeon Peas | 17.00 | 5 | Tomatoes | 5 |
|  | Cow Peas, dry | 16.00 | 5 | Cantaloupes & oth. melons | 95 |
|  | Cantaloupes & oth. melons | 16.00 | 95 | Sorghum | 0 |
|  | Peas, dry | 15.00 | 0 | Sunflowerseed | 25 |
|  | Soybeans | 15.00 | 25 | Chillies and peppers, dry | 5 |
|  | Green Corn (Maize) | 15.00 | 0 | Beans, dry | 5 |
|  | Broad Beans, dry | 13.00 | 5 | Chick-Peas | 0 |
|  | Beans, dry | 12.00 | 5 | Peas, dry | 0 |
|  | Beans, green | 12.00 | 5 | Chillies & Peppers, green | 5 |
|  | Cashew nuts, with shell | 12.00 | 65 | Millet | 0 |
| Zinc (mg) | Melonseed | 10.24 | 95 | Rice | 0 |
|  | Sesameseed | 7.75 | 25 | Wheat | 0 |
|  | Cashew nuts, with shell | 5.78 | 65 | Maize | 0 |
|  | Safflowerseed | 5.05 | 5 | Barley | 0 |
|  | Sunflowerseed | 5.00 | 25 | Soybeans | 25 |
|  | Lentils | 4.78 | 0 | Sunflowerseed | 25 |
|  | Rice | 4.52 | 0 | Groundnuts in shell | 5 |
|  | Brazil Nuts | 4.06 | 95 | Oats | 0 |
|  | Pulses nes | 4.00 | 5 | Rye | 0 |
|  | Oats | 3.97 | 0 | Coconuts | 25 |
|  | Rye | 3.73 | 0 | Beans, dry | 5 |
|  | Triticale | 3.45 | 0 | Millet | 0 |
|  | Chick-Peas | 3.43 | 0 | Triticale | 0 |
|  | Cow Peas, dry | 3.37 | 5 | Peas, dry | 0 |
|  | Groundnuts in shell | 3.27 | 5 | Chick-Peas | 0 |
|  | Wheat | 3.25 | 0 | Sesameseed | 25 |
|  | Broad Beans, dry | 3.14 | 5 | Tomatoes | 5 |
|  | Beans, dry | 3.10 | 5 | Beans, green | 5 |
|  | Beans, green | 3.10 | 5 | Lentils | 0 |
|  | Quinoa | 3.10 | 0 | Pulses nes | 5 |
| Copper (mg) | Sesameseed | 4.08 | 25 | Wheat | 0 |
|  | Cashew nuts, with shell | 2.20 | 65 | Maize | 0 |
|  | Sunflowerseed | 1.80 | 25 | Rice | 0 |
|  | Safflowerseed | 1.75 | 5 | Barley | 0 |
|  | Brazil Nuts | 1.74 | 95 | Sunflowerseed | 25 |
|  | Hazelnuts in shell | 1.73 | 0 | Groundnuts in shell | 5 |
|  | Pulses nes | 1.64 | 5 | Soybeans | 25 |
|  | Walnuts | 1.59 | 0 | Coconuts | 25 |
|  | Pistachios | 1.30 | 0 | Millet | 0 |
|  | Groundnuts in shell | 1.14 | 5 | Oats | 0 |
|  | Buckwheat | 1.10 | 65 | Beans, dry | 5 |
|  | Pigeon Peas | 1.06 | 5 | Sesameseed | 25 |
|  | Almonds, with shell | 1.00 | 65 | Peas, dry | 0 |
|  | Beans, dry | 0.87 | 5 | Rye | 0 |
|  | Beans, green | 0.87 | 5 | Grapes | 0 |
|  | Peas, dry | 0.87 | 0 | Chick-Peas | 0 |
|  | Chick-Peas | 0.85 | 0 | Tomatoes | 5 |
|  | Cow Peas, dry | 0.85 | 5 | Pulses nes | 5 |
|  | Broad Beans, dry | 0.82 | 5 | Cashew nuts, with shell | 65 |
|  | Nuts nes | 0.78 | 18.33 | Beans, green | 5 |
| Manganese (mg) | Hazelnuts in shell | 6.18 | 0 | Rice | 0 |
|  | Oats | 4.92 | 0 | Wheat | 0 |
|  | Nuts nes | 3.75 | 18.33 | Maize | 0 |
|  | Rice | 3,74 | 0 | Barley | 0 |
|  | Wheat | 3.72 | 0 | Oats | 0 |
|  | Walnuts | 3.41 | 0 | Soybeans | 25 |
|  | Triticale | 3.21 | 0 | Coconuts | 25 |
|  | Rye | 2.68 | 0 | Groundnuts in shell | 5 |
|  | Sesameseed | 2.46 | 25 | Sunflowerseed | 25 |
|  | Almonds, with shell | 2.29 | 65 | Rye | 0 |
|  | Chick-Peas | 2.20 | 0 | Millet | 0 |
|  | Quinoa | 2.03 | 0 | Triticale | 0 |
|  | Safflowerseed | 2.01 | 5 | Beans, dry | 5 |
|  | Sunflowerseed | 1.95 | 25 | Chick-Peas | 0 |
|  | Groundnuts in shell | 1.93 | 5 | Peas, dry | 0 |
|  | Pigeon Peas | 1.79 | 5 | Tomatoes | 5 |
|  | Cashew nuts, with shell | 1.66 | 65 | Spinach | 0 |
|  | Millet | 1.63 | 0 | Sesameseed | 25 |
|  | Broad Beans, dry | 1.63 | 5 | Beans, green | 5 |
|  | Melonseed | 1.61 | 95 | Eggplants | 25 |
| Selenium (mg) | Brazil Nuts | 1917.00 | 95 | Wheat | 0 |
|  | Wheat | 80.05 | 0 | Rice | 0 |
|  | Sunflowerseed | 53.00 | 25 | Maize | 0 |
|  | Barley | 37.70 | 0 | Barley | 0 |
|  | Rye | 35.30 | 0 | Sunflowerseed | 25 |
|  | Rice | 23.40 | 0 | Rye | 0 |
|  | Cashew nuts, with shell | 19.90 | 65 | Coconuts | 25 |
|  | Maize | 15.50 | 0 | Soybeans | 25 |
|  | Coconuts | 10.10 | 25 | Groundnuts in shell | 5 |
|  | Cow Peas, dry | 9.00 | 5 | Beans, dry | 5 |
|  | Quinoa | 8.50 | 0 | Brazil Nuts | 95 |
|  | Lentils | 8.30 | 0 | Millet | 0 |
|  | Buckwheat | 8.30 | 65 | Chick-Peas | 0 |
|  | Chick-Peas | 8.20 | 0 | Cashew nuts, with shell | 65 |
|  | Pigeon Peas | 8.20 | 5 | Beans, green | 5 |
|  | Broad Beans, dry | 8.20 | 5 | Cow Peas, dry | 5 |
|  | Beans, dry | 7.58 | 5 | Broad Beans, dry | 5 |
|  | Beans, green | 7.58 | 5 | Watermelons | 95 |
|  | Groundnuts in shell | 7.20 | 5 | Oranges | 5 |
|  | Pistachios | 7.00 | 0 | Lentils | 0 |
| Vitamin C (mg) | Fruit Fresh nes | 256.53 | 35.38 | Chillies & Peppers, green | 5 |
|  | Chillies & Peppers, green | 242.50 | 5 | Soybeans | 25 |
|  | Currants | 111.00 | 25 | Fruit Fresh nes | 35.38 |
|  | Kiwi Fruit | 92.70 | 95 | Oranges | 5 |
|  | Papayas | 61.80 | 5 | Tomatoes | 5 |
|  | Strawberries | 58.80 | 25 | Cantaloupes & oth. melons | 95 |
|  | Oranges | 53.20 | 5 | Tangerines and clementines | 5 |
|  | Lemons and limes | 41.05 | 5 | Mangoes | 65 |
|  | Peas, green | 40.00 | 0 | Grapes | 0 |
|  | Tangerines and clementines | 37.75 | 5 | Watermelons | 95 |
|  | Fruit Tropical Fresh nes | 37.17 | 43.93 | Lemons and limes | 5 |
|  | Cantaloupes & oth. melons | 36.70 | 95 | Fruit Tropical Fresh nes | 43.93 |
|  | Chestnuts | 36.00 | 25 | Papayas | 5 |
|  | Grapefruit and Pomelos | 33.30 | 5 | Peas, green | 0 |
|  | Chillies and peppers, dry | 31.40 | 5 | Spinach | 0 |
|  | Soybeans | 29.00 | 25 | Apples | 65 |
|  | Spinach | 28.10 | 0 | Pumpkins, squash & gourds | 95 |
|  | Mangoes | 27.70 | 65 | Strawberries | 25 |
|  | Blackberries and Raspberries | 23.60 | 65 | Coconuts | 25 |
|  | Okra | 21.10 | 25 | Grapefruit and Pomelos | 5 |
| Thiamin | Wheat | 6.31 | 0 | Wheat | 0 |
| (mg) | Sunflowerseed | 1.48 | 25 | Maize | 0 |
|  | Safflowerseed | 1.16 | 5 | Rice | 0 |
|  | Lentils | 0.87 | 0 | Soybeans | 25 |
|  | Pistachios | 0.87 | 0 | Oil palm fruit | 5 |
|  | Cow Peas, dry | 0.85 | 5 | Sunflowerseed | 25 |
|  | Sesameseed | 0.79 | 25 | Barley | 0 |
|  | Oats | 0.76 | 0 | Groundnuts in shell | 5 |
|  | Pulses nes | 0.73 | 5 | Oats | 0 |
|  | Peas, dry | 0.73 | 0 | Sorghum | 0 |
|  | Beans, dry | 0.71 | 5 | Beans, dry | 5 |
|  | Beans, green | 0.71 | 5 | Millet | 0 |
|  | Hazelnuts in shell | 0.64 | 0 | Peas, dry | 0 |
|  | Pigeon Peas | 0.64 | 5 | Rye | 0 |
|  | Groundnuts in shell | 0.64 | 5 | Oranges | 5 |
|  | Brazil Nuts | 0.62 | 95 | Triticale | 0 |
|  | Nuts nes | 0.56 | 18.33 | Grapes | 0 |
|  | Broad Beans, dry | 0.56 | 5 | Beans, green | 5 |
|  | Chick-Peas | 0.48 | 0 | Tomatoes | 5 |
|  | Soybeans | 0.44 | 25 | Chick-Peas | 0 |
| Riboflavin (mg) | Wheat | 6.09 | 0 | Wheat | 0 |
|  | Chillies and peppers, dry | 1.21 | 5 | Maize | 0 |
|  | Almonds, with shell | 1.01 | 65 | Rice | 0 |
|  | Buckwheat | 0.43 | 65 | Soybeans | 25 |
|  | Safflowerseed | 0.42 | 5 | Oil palm fruit | 5 |
|  | Sunflowerseed | 0.36 | 25 | Barley | 0 |
|  | Broad Beans, dry | 0.33 | 5 | Sunflowerseed | 25 |
|  | Quinoa | 0.32 | 0 | Millet | 0 |
|  | Millet | 0.29 | 0 | Sorghum | 0 |
|  | Rye | 0.25 | 0 | Groundnuts in shell | 5 |
|  | Sesameseed | 0.25 | 25 | Rye | 0 |
|  | Cow Peas, dry | 0.23 | 5 | Grapes | 0 |
|  | Peas, dry | 0.22 | 0 | Beans, dry | 5 |
|  | Chick-Peas | 0.21 | 0 | Oats | 0 |
|  | Lentils | 0.21 | 0 | Chillies and peppers, dry | 5 |
|  | Pulses nes | 0.21 | 5 | Fruit Fresh nes | 35.38 |
|  | Beans, dry | 0.20 | 5 | Oranges | 5 |
|  | Beans, green | 0.20 | 5 | Peas, dry | 0 |
|  | Maize | 0.20 | 0 | Tomatoes | 5 |
|  | Spinach | 0.19 | 0 | Spinach | 0 |
| Niacin (mg) | Groundnuts in shell | 12.07 | 5 | Wheat | 0 |
|  | Wheat | 9.88 | 0 | Rice | 0 |
|  | Chillies and peppers, dry | 8.67 | 5 | Maize | 0 |
|  | Sunflowerseed | 8.34 | 25 | Barley | 0 |
|  | Buckwheat | 7.02 | 65 | Groundnuts in shell | 5 |
|  | Millet | 4.72 | 0 | Soybeans | 25 |
|  | Rice | 4.70 | 0 | Oil palm fruit | 5 |
|  | Barley | 4.60 | 0 | Sunflowerseed | 25 |
|  | Sesameseed | 4.52 | 25 | Sorghum | 0 |
|  | Rye | 4.27 | 0 | Millet | 0 |
|  | Maize | 3.63 | 0 | Rye | 0 |
|  | Melonseed | 3.55 | 95 | Tomatoes | 5 |
|  | Almonds, with shell | 3.39 | 65 | Beans, dry | 5 |
|  | Pigeon Peas | 2.97 | 5 | Peas, dry | 0 |
|  | Sorghum | 2.93 | 0 | Coconuts | 25 |
|  | Peas, dry | 2.89 | 0 | Oats | 0 |
|  | Broad Beans, dry | 2.83 | 5 | Carrots & Turnips | 65 |
|  | Lentils | 2.61 | 0 | Chillies and peppers, dry | 5 |
|  | Safflowerseed | 2.28 | 5 | Eggplants | 25 |
|  | Nuts nes | 2.13 | 18.33 | Buckwheat | 65 |
| Pantothenic acid (mg) | Safflowerseed | 4.03 | 5 | Rice | 0 |
|  | Lentils | 2.14 | 0 | Wheat | 0 |
|  | Groundnuts in shell | 1.77 | 5 | Maize | 0 |
|  | Peas, dry | 1.76 | 0 | Groundnuts in shell | 5 |
|  | Chick-Peas | 1.59 | 0 | Barley | 0 |
|  | Cow Peas, dry | 1.50 | 5 | Oats | 0 |
|  | Rice | 1.49 | 0 | Sunflowerseed | 25 |
|  | Avocados | 1.46 | 65 | Soybeans | 25 |
|  | Rye | 1.46 | 0 | Rye | 0 |
|  | Oats | 1.35 | 0 | Millet | 0 |
|  | Triticale | 1.32 | 0 | Peas, dry | 0 |
|  | Pigeon Peas | 1.27 | 5 | Watermelons | 95 |
|  | Buckwheat | 1.23 | 65 | Beans, dry | 5 |
|  | Sunflowerseed | 1.13 | 25 | Coconuts | 25 |
|  | Broad Beans, dry | 0.98 | 5 | Oranges | 5 |
|  | Chillies and peppers, dry | 0.96 | 5 | Triticale | 0 |
|  | Hazelnuts in shell | 0.92 | 0 | Chick-Peas | 0 |
|  | Beans, dry | 0.88 | 5 | Tomatoes | 5 |
|  | Beans, green | 0.88 | 5 | Peaches and nectarines | 65 |
|  | Cashew nuts, with shell | 0.86 | 65 | Cucumbers and Gherkins | 65 |
| Vitamin B6 (mg) | Wheat | 6.52 | 0 | Wheat | 0 |
|  | Pistachios | 1.70 | 0 | Maize | 0 |
|  | Sunflowerseed | 1.35 | 25 | Rice | 0 |
|  | Safflowerseed | 1.17 | 5 | Barley | 0 |
|  | Chillies and peppers, dry | 0.81 | 5 | Sunflowerseed | 25 |
|  | Sesameseed | 0.79 | 25 | Soybeans | 25 |
|  | Maize | 0.62 | 0 | Groundnuts in shell | 5 |
|  | Hazelnuts in shell | 0.56 | 0 | Millet | 0 |
|  | Lentils | 0.54 | 0 | Tomatoes | 5 |
|  | Walnuts | 0.54 | 0 | Beans, dry | 5 |
|  | Chick-Peas | 0.54 | 0 | Chillies & Peppers, green | 5 |
|  | Rice | 0.51 | 0 | Grapes | 0 |
|  | Quinoa | 0.49 | 0 | Rye | 0 |
|  | Cashew nuts, with shell | 0.42 | 65 | Chick-Peas | 0 |
|  | Chestnuts | 0.41 | 25 | Watermelons | 95 |
|  | Beans, dry | 0.39 | 5 | Oranges | 5 |
|  | Beans, green | 0.39 | 5 | Mangoes | 65 |
|  | Millet | 0.38 | 0 | Carrots & Turnips | 65 |
|  | Broad Beans, dry | 0.37 | 5 | Fruit Fresh nes | 35.38 |
|  | Cow Peas, dry | 0.36 | 5 | Oats | 0 |
| Folate (mcg) | Cow Peas, dry | 633.00 | 5 | Soybeans | 25 |
|  | Chick-Peas | 557.00 | 0 | Wheat | 0 |
|  | Lentils | 479.00 | 0 | Maize | 0 |
|  | Pigeon Peas | 456.00 | 5 | Rice | 0 |
|  | Broad Beans, dry | 423.00 | 5 | Groundnuts in shell | 5 |
|  | Beans, dry | 421.00 | 5 | Beans, dry | 5 |
|  | Beans, green | 421.00 | 5 | Sunflowerseed | 25 |
|  | Peas, dry | 274.00 | 0 | Chick-Peas | 0 |
|  | Groundnuts in shell | 240.00 | 5 | Barley | 0 |
|  | Sunflowerseed | 227.00 | 25 | Peas, dry | 0 |
|  | Spinach | 194.00 | 0 | Cow Peas, dry | 5 |
|  | Quinoa | 184.00 | 0 | Beans, green | 5 |
|  | Soybeans | 165.00 | 25 | Millet | 0 |
|  | String beans | 162.67 | 5 | Spinach | 0 |
|  | Safflowerseed | 160.00 | 5 | Oranges | 5 |
|  | Hazelnuts in shell | 113.00 | 0 | Broad Beans, dry | 5 |
|  | Walnuts | 98.00 | 0 | Tomatoes | 5 |
|  | Sesameseed | 97.00 | 25 | Lentils | 0 |
|  | Avocados | 89.00 | 65 | Pigeon Peas | 5 |
|  | Okra | 88.00 | 25 | Oats | 0 |
| Vitamin A (IU) | Chillies and peppers, dry | 26488.00 | 5 | Carrots & Turnips | 65 |
|  | Carrots & Turnips | 16811.00 | 65 | Maize | 0 |
|  | Spinach | 9377.00 | 0 | Spinach | 0 |
|  | Cantaloupes & oth. melons | 3382.00 | 95 | Tomatoes | 5 |
|  | Pumpkins, squash & gourds | 2983.67 | 95 | Cantaloupes & oth. melons | 95 |
|  | Apricots | 1926.00 | 65 | Chillies and peppers, dry | 5 |
|  | Persimmons | 1627.00 | 5 | Pumpkins, squash & gourds | 95 |
|  | Sour Cherries | 1283.00 | 65 | Watermelons | 95 |
|  | Chillies & Peppers, green | 1179.00 | 5 | Soybeans | 25 |
|  | Papayas | 1094.00 | 5 | Chillies & Peppers, green | 5 |
|  | Tomatoes | 833.00 | 5 | Mangoes | 65 |
|  | Fruit Fresh nes | 817.00 | 35.38 | Fruit Fresh nes | 35.38 |
|  | Peas, green | 765.00 | 0 | Tangerines and clementines | 5 |
|  | Mangoes | 765.00 | 65 | Oranges | 5 |
|  | Tangerines and clementines | 681.00 | 5 | Papayas | 5 |
|  | Watermelons | 569.00 | 95 | Olives | 0 |
|  | Pistachios | 553.00 | 0 | Peas, green | 0 |
|  | String beans | 520.33 | 5 | Apricots | 65 |
|  | Olives | 403.00 | 0 | Peaches and nectarines | 65 |
|  | Okra | 375.00 | 25 | Persimmons | 5 |
| Vitamin E (mg) | Sunflowerseed | 33.23 | 25 | Sunflowerseed | 25 |
|  | Almonds, with shell | 26.22 | 65 | Rice | 0 |
|  | Hazelnuts in shell | 15.03 | 0 | Wheat | 0 |
|  | Groundnuts in shell | 8.33 | 5 | Maize | 0 |
|  | Brazil Nuts | 5.73 | 95 | Groundnuts in shell | 5 |
|  | Nuts nes | 3.76 | 18.33 | Tomatoes | 5 |
|  | Chillies and peppers, dry | 3.14 | 5 | Almonds, with shell | 65 |
|  | Quinoa | 2.44 | 0 | Fruit Fresh nes | 35.38 |
|  | Pistachios | 2.30 | 0 | Mangoes | 65 |
|  | Spinach | 2.03 | 0 | Olives | 0 |
|  | Avocados | 1.97 | 65 | Rye | 0 |
|  | Fruit Fresh nes | 1.65 | 35.38 | Spinach | 0 |
|  | Olives | 1.65 | 0 | Chillies & Peppers, green | 5 |
|  | Kiwi Fruit | 1.46 | 95 | Carrots & Turnips | 65 |
|  | Rye | 1.28 | 0 | Coconuts | 25 |
|  | Cranberries | 1.20 | 65 | Grapes | 0 |
|  | Rice | 1.20 | 0 | Hazelnuts in shell | 0 |
|  | Mangoes | 1.12 | 65 | Peaches and nectarines | 65 |
|  | Blackberries and Raspberries | 1.02 | 65 | Oranges | 5 |
|  | Wheat | 1.01 | 0 | Apples | 65 |
| β-Tocopherol | Sunflowerseed | 1.14 | 25 | Sunflowerseed | 25 |
| (mg) | Hazelnuts in shell | 0.33 | 0 | Tomatoes | 5 |
|  | Almonds, with shell | 0.29 | 65 | Fruit Fresh nes | 35.38 |
|  | Walnuts | 0.15 | 0 | Peaches and nectarines | 65 |
|  | Nuts nes | 0.13 | 18.33 | Almonds, with shell | 65 |
|  | Quinoa | 0.08 | 0 | Cucumbers and Gherkins | 65 |
|  | Fruit Fresh nes | 0.05 | 35.38 | Hazelnuts in shell | 0 |
|  | Blackberries and Raspberries | 0.05 | 65 | Carrots & Turnips | 65 |
|  | Peaches and nectarines | 0.05 | 65 | Walnuts | 0 |
|  | Avocados | 0.04 | 65 | Avocados | 65 |
|  | Cashew nuts, with shell | 0.03 | 65 | Nuts nes | 18.33 |
|  | Carrots & Turnips | 0.01 | 65 | Cashew nuts, with shell | 65 |
|  | Blueberries | 0.01 | 65 | Strawberries | 25 |
|  | Tomatoes | 0.01 | 5 | Blackberries and Raspberries | 65 |
|  | Strawberries | 0.01 | 25 | Cherries | 65 |
|  | Cherries | 0.01 | 65 | Quinoa | 0 |
|  | Cucumbers and Gherkins | 0.01 | 65 | Blueberries | 65 |
| γ - Tocopherol | Pistachios | 22.60 | 0 | Beans, dry | 5 |
| (mg) | Walnuts | 20.83 | 0 | Walnuts | 0 |
|  | Nuts nes | 11.86 | 18.33 | Coconuts | 25 |
|  | Brazil Nuts | 7.87 | 95 | Fruit Fresh nes | 35.38 |
|  | Cashew nuts, with shell | 5.31 | 65 | Peas, dry | 0 |
|  | Quinoa | 4.55 | 0 | Lentils | 0 |
|  | Lentils | 4.23 | 0 | Tomatoes | 5 |
|  | Peas, dry | 2.09 | 0 | Cashew nuts, with shell | 65 |
|  | Beans, dry | 2.01 | 5 | Beans, green | 5 |
|  | Beans, green | 2.01 | 5 | Pistachios | 0 |
|  | Blackberries and Raspberries | 1.38 | 65 | Nuts nes | 18.33 |
|  | Fruit Fresh nes | 1.34 | 35.38 | Peas, green | 0 |
|  | Peas, green | 0.95 | 0 | Grapes | 0 |
|  | Almonds, with shell | 0.65 | 65 | Sunflowerseed | 25 |
|  | Coconuts | 0.53 | 25 | Cantaloupes & oth. melons | 95 |
|  | Blueberries | 0.36 | 65 | Spinach | 0 |
|  | Avocados | 0.32 | 65 | Green Corn (Maize) | 0 |
|  | Spinach | 0.18 | 0 | Cucumbers and Gherkins | 65 |
|  | Green Corn (Maize) | 0.15 | 0 | Almonds, with shell | 65 |
|  | Sunflowerseed | 0.13 | 25 | Avocados | 65 |
| δ-Tocopherol (mg) | Walnuts | 1.89 | 0 | Beans, dry | 5 |
|  | Blackberries and Raspberries | 0.97 | 65 | Walnuts | 0 |
|  | Pistachios | 0.80 | 0 | Fruit Fresh nes | 35.38 |
|  | Brazil Nuts | 0.77 | 95 | Beans, green | 5 |
|  | Cashew nuts, with shell | 0.36 | 65 | Peas, dry | 0 |
|  | Quinoa | 0.35 | 0 | Cashew nuts, with shell | 65 |
|  | Beans, dry | 0.17 | 5 | Peaches and nectarines | 65 |
|  | Beans, green | 0.17 | 5 | Blackberries and Raspberries | 65 |
|  | Nuts nes | 0.16 | 18.33 | Pistachios | 0 |
|  | Fruit Fresh nes | 0.14 | 35.38 | Peas, green | 0 |
|  | Peas, dry | 0.09 | 0 | Nuts nes | 18.33 |
|  | Almonds, with shell | 0.05 | 65 | Almonds, with shell | 65 |
|  | Peaches and nectarines | 0.05 | 65 | Avocados | 65 |
|  | Blueberries | 0.03 | 65 | Brazil Nuts | 95 |
|  | Peas, green | 0.02 | 0 | Strawberries | 25 |
|  | Avocados | 0.02 | 65 | Quinoa | 0 |
|  | Strawberries | 0.01 | 25 | Blueberries | 65 |
| Fluoride (mcg) | Almonds, with shell | 1692562.60 | 65 | Almonds, with shell | 65 |
|  | String beans | 19.00 | 5 | Grapes | 0 |
|  | Nuts nes | 10.00 | 18.33 | Tomatoes | 5 |
|  | Grapes | 7.80 | 0 | Apples | 65 |
|  | Strawberries | 4.40 | 25 | Watermelons | 95 |
|  | Peaches and nectarines | 4.00 | 65 | Peaches and nectarines | 65 |
|  | Apples | 3.30 | 65 | Cucumbers and Gherkins | 65 |
|  | Tomatoes | 2.30 | 5 | Pears | 65 |
|  | Pears | 2.20 | 65 | String beans | 5 |
|  | Plums and sloes | 2.00 | 65 | Cantaloupes & oth. melons | 95 |
|  | Cherries | 2.00 | 65 | Plums and sloes | 65 |
|  | Watermelons | 1.50 | 95 | Strawberries | 25 |
|  | Cucumbers and Gherkins | 1.30 | 65 | Nuts nes | 18.33 |
|  | Cantaloupes & oth. melons | 1.00 | 95 | Cherries | 65 |
| Vitamin K (mcg) | Spinach | 482.90 | 0 | Spinach | 0 |
|  | Chillies and peppers, dry | 108.20 | 5 | Rice | 0 |
|  | Okra | 53.00 | 25 | Wheat | 0 |
|  | Kiwi Fruit | 40.30 | 95 | Grapes | 0 |
|  | Cashew nuts, with shell | 34.10 | 65 | Tomatoes | 5 |
|  | Nuts nes | 28.70 | 18.33 | Cucumbers and Gherkins | 65 |
|  | Peas, green | 24.80 | 0 | Chillies & Peppers, green | 5 |
|  | Avocados | 21.00 | 65 | Carrots & Turnips | 65 |
|  | Blueberries | 19.30 | 65 | Barley | 0 |
|  | Cucumbers and Gherkins | 16.40 | 65 | Okra | 25 |
|  | Grapes | 14.60 | 0 | Chillies and peppers, dry | 5 |
|  | Peas, dry | 14.50 | 0 | Fruit Fresh nes | 35.38 |
|  | String beans | 14.40 | 5 | Maize | 0 |
|  | Chillies & Peppers, green | 14.30 | 5 | Peas, green | 0 |
|  | Hazelnuts in shell | 14.20 | 0 | Peas, dry | 0 |
|  | Blackberries and Raspberries | 13.80 | 65 | Beans, dry | 5 |
|  | Carrots & Turnips | 13.20 | 65 | Apples | 65 |
|  | Fruit Fresh nes | 11.38 | 35.38 | Mangoes | 65 |
|  | Chick-Peas | 9.00 | 0 | Rye | 0 |
|  | Broad Beans, dry | 9.00 | 5 | Eggplants | 25 |
| β-Carotene | Chillies and peppers, dry | 14844.00 | 5 | Carrots & Turnips | 65 |
| (mcg) | Carrots & Turnips | 8285.00 | 65 | Maize | 0 |
|  | Spinach | 5626.00 | 0 | Spinach | 0 |
|  | Cantaloupes & oth. melons | 2020.00 | 95 | Tomatoes | 5 |
|  | Pumpkins, squash & gourds | 1346.67 | 95 | Cantaloupes & oth. melons | 95 |
|  | Apricots | 1094.00 | 65 | Chillies and peppers, dry | 5 |
|  | Sour Cherries | 770.00 | 65 | Pumpkins, squash & gourds | 95 |
|  | Chillies & Peppers, green | 671.00 | 5 | Watermelons | 95 |
|  | Fruit Fresh nes | 478.60 | 35.38 | Chillies & Peppers, green | 5 |
|  | Peas, green | 449.00 | 0 | Mangoes | 65 |
|  | Tomatoes | 449.00 | 5 | Fruit Fresh nes | 35.38 |
|  | Mangoes | 445.00 | 65 | Oranges | 5 |
|  | String beans | 379.00 | 5 | Olives | 0 |
|  | Pistachios | 332.00 | 0 | Peas, green | 0 |
|  | Watermelons | 303.00 | 95 | Tangerines and clementines | 5 |
|  | Papayas | 276.00 | 5 | Apricots | 65 |
|  | Persimmons | 253.00 | 5 | Wheat | 0 |
|  | Olives | 237.00 | 0 | Grapes | 0 |
|  | Okra | 225.00 | 25 | Peaches and nectarines | 65 |
|  | Plums and sloes | 190.00 | 65 | Fruit Tropical Fresh nes | 43.93 |
| α-Carotene | Carrots & Turnips | 3477.00 | 65 | Carrots & Turnips | 65 |
| (mcg) | Chillies and peppers, dry | 994.00 | 5 | Maize | 0 |
|  | Pumpkins, squash & gourds | 171.67 | 95 | Tomatoes | 5 |
|  | Tomatoes | 101.00 | 5 | Pumpkins, squash & gourds | 95 |
|  | Tangerines and clementines | 101.00 | 5 | Chillies and peppers, dry | 5 |
|  | String beans | 69.00 | 5 | Tangerines and clementines | 5 |
|  | Maize | 63.00 | 0 | Oranges | 5 |
|  | Avocados | 24.00 | 65 | Chillies & Peppers, green | 5 |
|  | Chillies & Peppers, green | 23.00 | 5 | Mangoes | 65 |
|  | Peas, green | 21.00 | 0 | Cucumbers and Gherkins | 65 |
|  | Apricots | 19.00 | 65 | Cantaloupes & oth. melons | 95 |
|  | Mangoes | 17.00 | 65 | Peas, green | 0 |
|  | Cantaloupes & oth. melons | 16.00 | 95 | String beans | 5 |
|  | Oranges | 11.00 | 5 | Fruit Fresh nes | 35.38 |
|  | Cucumbers and Gherkins | 11.00 | 65 | Green Corn (Maize) | 0 |
|  | Green Corn (Maize) | 9.00 | 0 | Avocados | 65 |
|  | Blackberries and Raspberries | 8.00 | 5 | Grapes | 0 |
|  | Grapefruit and Pomelos | 8.00 | 65 | Apricots | 65 |
|  | Fruit Fresh nes | 6.20 | 35.38 | Fruit Tropical Fresh nes | 43.93 |
|  | Fruit Tropical Fresh nes | 3.88 | 43.93 | Grapefruit and Pomelos | 5 |
| β-Crytoxanthin (mcg) | Persimmons | 1447.00 | 5 | Pumpkins, squash & gourds | 95 |
|  | Chillies and peppers, dry | 1103.00 | 5 | Tangerines and clementines | 5 |
|  | Papayas | 761.00 | 5 | Oranges | 5 |
|  | Pumpkins, squash & gourds | 715.00 | 95 | Watermelons | 95 |
|  | Tangerines and clementines | 407.00 | 5 | Papayas | 5 |
|  | Carrots & Turnips | 125.00 | 65 | Persimmons | 5 |
|  | Oranges | 116.00 | 5 | Carrots & Turnips | 65 |
|  | Apricots | 104.00 | 65 | Chillies and peppers, dry | 5 |
|  | Fruit Fresh nes | 97.20 | 35.38 | Fruit Fresh nes | 35.38 |
|  | Peaches and nectarines | 82.50 | 65 | Peaches and nectarines | 65 |
|  | Watermelons | 78.00 | 95 | Chillies & Peppers, green | 5 |
|  | Green Corn (Maize) | 63.50 | 0 | Cucumbers and Gherkins | 65 |
|  | Chillies & Peppers, green | 50.00 | 5 | Apples | 65 |
|  | Plums and sloes | 35.00 | 65 | Green Corn (Maize) | 0 |
|  | Avocados | 27.00 | 65 | Plums and sloes | 65 |
|  | Cucumbers and Gherkins | 26.00 | 65 | Mangoes | 65 |
|  | Mangoes | 11.00 | 65 | Apricots | 65 |
|  | Apples | 11.00 | 65 | Olives | 0 |
|  | Lemons and limes | 10.00 | 5 | Lemons and limes | 5 |
|  | Olives | 9.00 | 0 | Avocados | 65 |
| Lycopene | Watermelons | 4532.00 | 95 | Watermelons | 95 |
| (mcg) | Tomatoes | 2573.00 | 5 | Tomatoes | 5 |
|  | Fruit Fresh nes | 1700.00 | 35.38 | Fruit Fresh nes | 35.38 |
|  | Fruit Tropical Fresh nes | 1301.00 | 43.93 | Fruit Tropical Fresh nes | 43.93 |
|  | Persimmons | 159.00 | 5 | Persimmons | 5 |
|  | Carrots & Turnips | 1.00 | 65 | Carrots & Turnips | 65 |
| Lutein & Zeaxanthin (mcg) | Spinach | 12198.00 | 0 | Maize | 0 |
|  | Chillies and peppers, dry | 5494.00 | 5 | Spinach | 0 |
|  | Peas, green | 2477.00 | 0 | Wheat | 0 |
|  | Maize | 1355.00 | 0 | Pumpkins, squash & gourds | 95 |
|  | Pumpkins, squash & gourds | 1221.00 | 95 | Barley | 0 |
|  | Persimmons | 834.00 | 5 | Peas, green | 0 |
|  | Chillies & Peppers, green | 725.00 | 5 | Chillies & Peppers, green | 5 |
|  | String beans | 640.00 | 5 | Tomatoes | 5 |
|  | Okra | 516.00 | 25 | Chillies and peppers, dry | 5 |
|  | Olives | 510.00 | 0 | Fruit Fresh nes | 35.38 |
|  | Fruit Fresh nes | 500.25 | 35.38 | Olives | 0 |
|  | Green Corn (Maize) | 399.00 | 0 | Oranges | 5 |
|  | Avocados | 271.00 | 65 | Carrots & Turnips | 65 |
|  | Carrots & Turnips | 256.00 | 65 | Grapes | 0 |
|  | Wheat | 220.00 | 0 | Rye | 0 |
|  | Rye | 210.00 | 0 | Green Corn (Maize) | 0 |
|  | Quinoa | 163.00 | 0 | Tangerines and clementines | 5 |
|  | Barley | 160.00 | 0 | Okra | 25 |
|  | Tangerines and clementines | 138.00 | 5 | Persimmons | 5 |
|  | Oranges | 129.00 | 5 | Apples | 65 |
